# Un lymphangiome microkystique

**DOI:** 10.11604/pamj.2014.19.374.3074

**Published:** 2014-12-12

**Authors:** Amal Akazane, Badreddine Hassam

**Affiliations:** 1 Service de Dermatologie-Vénérologie, CHU Ibn Sina, Rabat, Maroc; 2 Faculté de Médecine et de Pharmacie Med V Souissi, Rabat, Maroc

**Keywords:** Lymphangiome, kystiques, vésicules translucides, Lymphangioma, cystic, translucent vesicles

## Image en medicine

Les malformations lymphatiques kystiques cutanées sont des dilatations plus ou moins importantes développées à partir du système lymphatique. Elles sont macrokystiques, microkystiques ou mixtes. Les malformations microkystiques superficielles (lymphangioma circumscriptum) se manifestent par des groupements de vésicules translucides ou hématiques, des papules ou des plaques roses infiltrées ou hyperkératosiques Le plus souvent, les lésions sont présentes à la naissance ou bien elles débutent dans la première enfance. Les localisations, les plus fréquentes, sont les parties proximales des membres. L'affection reste, la plupart du temps, asymptomatique mais il est noté des épisodes inflammatoires ou infectieux voire des saignements par rupture des vésicules lymphatiques. L'histologie des lymphangiomes microkystiques correspond à des dilatations lymphatiques dermiques ou épidermiques. L’évolution peut être émaillée de poussées inflammatoires, une disparition spontanée est possible mais rare. Le choix du traitement de d'un lymphangiome dépend de sa forme clinique et de sa localisation. Pour les formes microkystiques, on peut discuter l'abstention, la sclérothérapie, l’électrocoagulation, le laser CO2, l'excision chirurgicale. Nous rapportons un cas de lymphangiome microkystique chez une patiente de 22 ans sans antécédant notable; qui présente une lésion vésiculeuse de la face interne du genou droit évoluant depuis l’âge de 2 ans associé à un prurit intermittent avec un suintement de liquide clair; L'examen clinique note une tuméfaction indolore de la face interne du genou droit; de 3 cm de grand axe surmonté de vésicules translusides et d'autres hématiques, l'histologie de la biopsie cutanée a confirmé le diagnostic de lymphangiome sous cutané en montrant un épiderme d’épaisseur normal orthokératosique soulevé par des formations vasculaires volumineuses comblées parfois par de la sérosité et bordées par des cellules endothéliales normales; ces formations vasculaires siègent dans les dermes papillaire et moyen ainsi une abstention thérapeutique a été préconisé.

**Figure 1:**
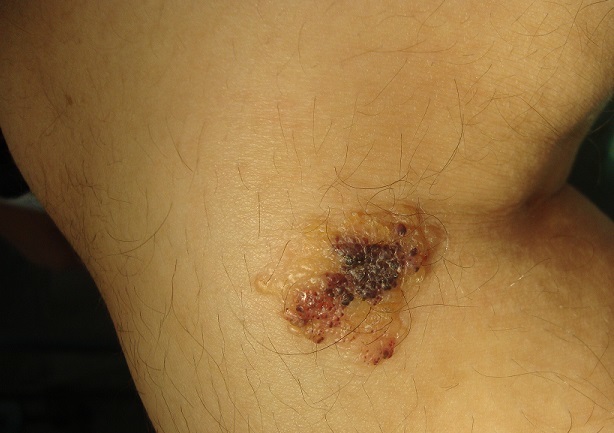
Groupement de vésicules translucides au niveau du genou

